# Small Non-coding RNAs Associated with Viral Infectious Diseases of Veterinary Importance: Potential Clinical Applications

**DOI:** 10.3389/fvets.2016.00022

**Published:** 2016-04-04

**Authors:** Mohamed Samir, Frank Pessler

**Affiliations:** ^1^TWINCORE Center for Experimental and Clinical Infection Research, Hannover, Germany; ^2^Zoonoses Department, Faculty of Veterinary Medicine, Zagazig University, Zagazig, Egypt; ^3^Helmholtz Center for Infection Research, Braunschweig, Germany

**Keywords:** animals, animal viruses, biomarkers, clinical application, infectious diseases, miRNA, small non-coding RNAs, veterinary science

## Abstract

MicroRNAs (miRNAs) represent a class of small non-coding RNA (sncRNA) molecules that can regulate mRNAs by inducing their degradation or by blocking translation. Considering that miRNAs are ubiquitous, stable, and conserved across animal species, it seems feasible to exploit them for clinical applications. Unlike in human viral diseases, where some miRNA-based molecules have progressed to clinical application, in veterinary medicine, this concept is just starting to come into view. Clinically, miRNAs could represent powerful diagnostic tools to pinpoint animal viral diseases and/or prognostic tools to follow up disease progression or remission. Additionally, the possible consequences of miRNA dysregulation make them potential therapeutic targets and open the possibilities to use them as tools to generate viral disease-resistant livestock. This review presents an update of preclinical studies on using sncRNAs to combat viral diseases that affect pet and farm animals. Moreover, we discuss the possibilities and challenges of bringing these bench-based discoveries to the veterinary clinic.

## Introduction

Small non-coding RNAs (sncRNAs) are classes of short RNAs, which do not encode proteins, but rather perform regulatory functions by engaging target transcripts through sequence-specific interactions. Among these, microRNAs (miRNAs) are single-stranded molecules roughly 22 nt in length (1). The regulatory network and function of miRNAs are based on the fact that more than one miRNA species can target the same mRNA (cooperativity) and that one miRNA can target hundreds of mRNA species (multiplicity) (2). The binding of miRNAs to the 3′ untranslated region (UTR) of particular mRNA leads to either mRNA degradation or protein translation repression (3). miRNAs can be highly regulated both in pattern and degree of expression across multiple animal diseases. Targeting hundreds of host and pathogen encoded genes, a single miRNA can influence the gene networks essential for development and progression of a disease condition (4). This, coupled with their high degree of conservation, has made miRNAs attractive candidates for clinical application to combat pathogenic animal viruses. Being highly stable, they can be used as disease biomarkers (5). The availability of chemically synthesized miRNA mimics and agonists and vector-based RNA interference (RNAi) technology raised the idea of therapies based on non-coding RNA and made it feasible to utilize this approach to create genetically modified animal breeds that are resistant to certain viral pathogens. In this review, we summarize the current state of laboratory studies geared toward clinical applications of sncRNAs [miRNAs, small interfering RNAs (siRNAs), and short hairpin RNAs (shRNAs)] to diagnose and combat viral diseases that affect animals of veterinary importance and may thus impact animal and human health.

## miRNAs as Candidates for Clinical Application to Combat Animal Viral Diseases

### Potential Biomarkers

The emerging correlation between miRNA expression and disease pathogenesis and outcomes suggests the potential use of miRNAs as biomarkers. In the first report that described the role of a miRNA as a diagnostic and prognostic marker in humans, Takamizawa et al. demonstrated that in patients with lung cancer, lower let-7 levels predicted a significantly worse prognosis after potentially curative resection (6). The intense use of advanced genomic technologies has resulted in rapid progress in human personalized medicine, where biomarker studies play a central role. Similar research interest has been emerging in veterinary medicine, albeit with some delay. Indeed, Henry et al. reported in 2010 that biomarker studies in veterinary medicine were still lagging behind those in humans (7). Biomarker research in the field of veterinary medicine focuses on the health and welfare of farm and companion animals as well as broader aspects, such as the biosafety of animal-derived food and milk production. Generally, the potential applications for biomarkers in veterinary clinics include diagnosis, staging, prognosis, and monitoring responses to therapy. Although several well-established biomarkers have been recognized for a number of veterinary viral diseases, there are still many barriers. As one example of many, lack of specificity has been recorded when using acute phase proteins (APPs) as biomarkers in pig, horse, and cattle suffering from inflammatory conditions that may have infectious etiologies, such as foot-and-mouth disease virus (FMDV) infection, porcine reproductive and respiratory syndrome virus infection, pneumonia, arthritis, enteritis, and post-castration inflammation (8–10). The concentration of some available biomarker molecules is affected by animal age (11). Thus, there is a need for additional, improved biomarkers for animal diseases. There is growing recognition that miRNAs may provide a specific signature that reflects the existence of a given clinical state. In this regard, the results of profiling the fluctuation of miRNA expression levels in infected organs, tissues, or single cells compared to uninfected ones throughout the course of a disease might reflect severity and outcome of the disease, including the likelihood of response to a given therapy. As biomarkers, miRNAs represent ideal candidates owing to their biological and clinical relevance, practicality, and consistent correlation with disease activity. The biological rational behind using miRNAs as biomarkers arises from their involvement in diverse physiological and pathological processes. miRNAs are extremely practical, with many advantages over other currently used biomarkers. For instance, there are efforts to develop them into diagnostics for the differentiation between viral and bacterial infections, each of which typically requires different interventions, such as quarantining versus feeding of antibiotics. The smaller number of identified miRNAs (12), compared to the approximately 30,000 protein-encoding genes currently known, implies that computational approaches dealing with miRNAs would be simpler and would require fewer resources than proteomics- or mRNA-based approaches. Another merit of miRNAs is their resistance to degradation by ribonucleases. For instance, they are stable in formalin-fixed, paraffin-embedded tissue (FFPE) independent of formalin fixation time and duration of tissue block storage (13). In contrast, mRNAs are highly fragmented and unstable in FFPE, which is problematic when FFPE is the only available sample type or when long storage of FFPE blocks has led to mRNA degradation (13). miRNAs can be detected in a large number of easily accessible samples, such as tissue biopsies, whole blood, blood cells, cerebrospinal fluid, saliva, urine, and other body fluids. Circulating miRNAs have proven to be highly resistant against RNAse activity, extreme pH, and temperature, and certainly more so than mRNAs. This is, at least in part, because they are often contained in lipid vesicles (microvesicles and exosomes) or bound by RNA-binding proteins (5). Additionally, miRNAs resist prolonged exposure to room temperature and repeated freezing/thawing cycles. Some miRNAs may be uniquely expressed only in specific body fluids, as exemplified by miR-224 (plasma/serum), miR-637 (tears), miR-193b (breast milk), and miR-508-5p (seminal fluid) (14). As opposed to miRNAs, proteins are a much more complex family of molecules due to use of alternate reading frames, splice variants, and various post-translational modifications, and many proteins of interest are of low abundance and/or may display major sequence variations among clinically relevant species (5).

#### MicroRNAs as Shared Biomarkers in Human and Animal Disease

**Figure 1:**
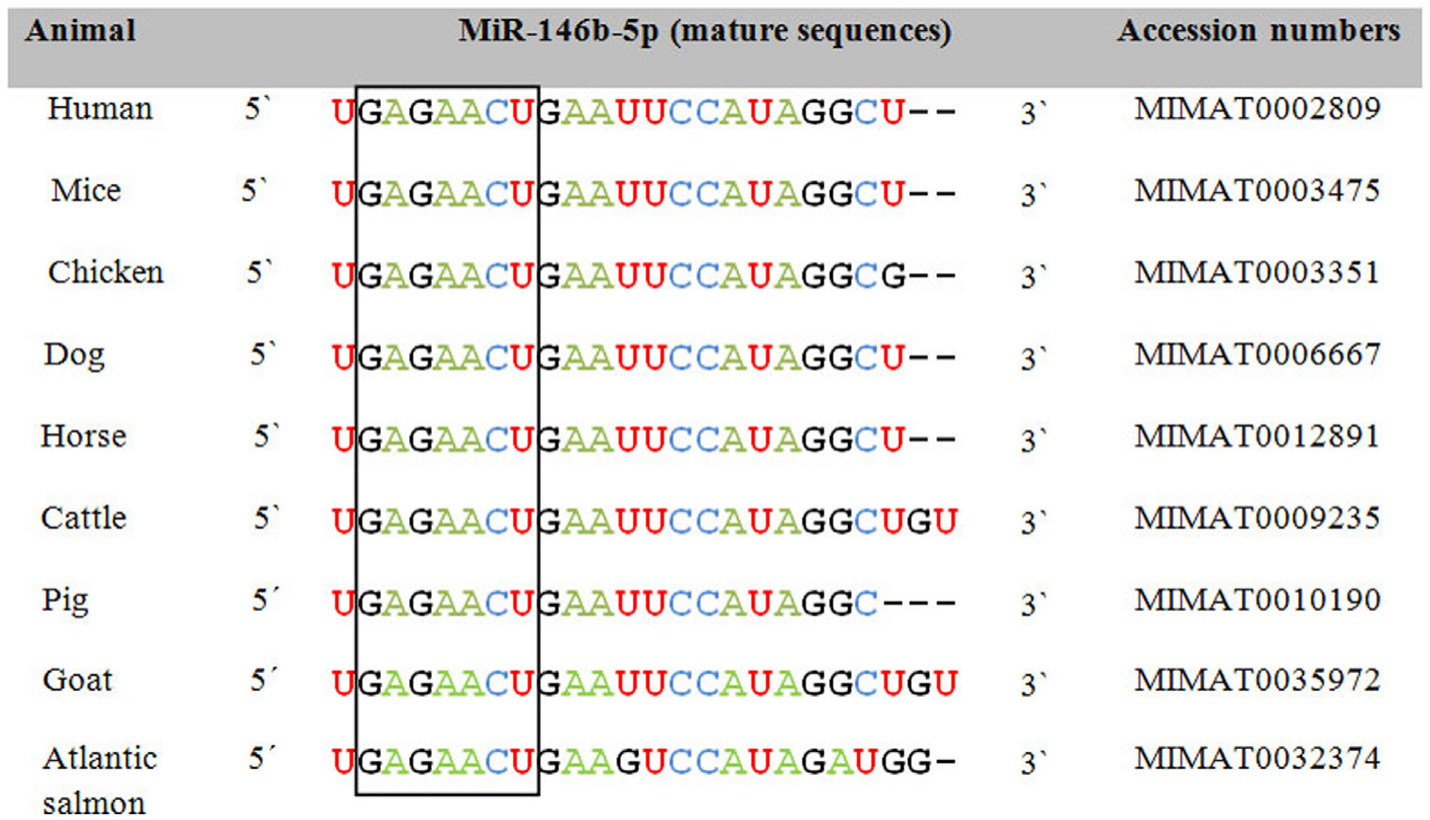
**Sequence alignment of the mature form of miR-146b-5p among animals and humans**. The open box illustrates the high degree of conservation of the seed region of miR-146b-5p. Accession numbers are according to miRBase 21 (12).

In order to assess the role of miRNAs as a class of shared biomarkers, it is important to investigate the cross-species conservation and regulation of the same miRNA species or miRNA family. Most of the annotated miRNAs are evolutionarily conserved among a variety of organisms, particularly in their mature form, suggesting that the majority of miRNAs constitute a large class of predominantly orthologous or homologous molecules. As exemplified by miR-146b-5p, cross-species variation in miRNA sequence is typically observed in 1 or 2 nt in the periphery of the mature form and in its 3′ UTR, i.e., away from the highly conserved seed region (Figure 1). This unique conservation pattern might be attributed to the conservation of their genomic origin. It has been reported that a consensus motif of 7–8 nt upstream and downstream of the pre-miRNA hairpin was found to be conserved among nematodes (15). Researchers from Slovenia and the USA have put together a catalog to describe the integrated assembly of intragenic miRNAs and their host genes in humans, mouse, and chicken (16). They showed that several miRNA genes were located within homologous areas, which implies that miRNA colocalization, co-expression, and potential coregulation may be conserved broadly across evolution and thus be applicable to both animal and human diseases. In the same context, previous studies reported that 300 canine miRNAs are homologs of annotated human miRNAs and that miRNA clusters are usually conserved between humans and dogs (17). Using next generation sequencing, Li et al. indicated that miRNAs in immune organs of chicken and duck were about 99% conserved (18). To gain further insights into miRNAs that are shared between species and might be used as common biomarkers, we selected a group of miRNAs that are commonly expressed upon influenza A virus (IAV) infection in humans and chicken. These miRNAs were further analyzed with miRviewer (19), a database that includes all known miRNAs of currently annotated animal genomes. William Pearson’s aligning program was used to assess the degree of conservation of mature miRNAs between the two species (Table 1). The percentage of sequence identity was further confirmed by the Bioedit sequence alignment editor (20). Indeed, there is a high degree of conservation of most miRNAs between the two species (Table 1). For some miRNAs, there are sequence differences between humans and chicken in the form of deletion or addition of extra nucleotides, but these are mostly located outside the seed region. We speculate that conserved miRNAs might be the most promising candidates for universal biomarkers that may help in simultaneously pinpointing a given disease state in both species. In contrast, the non-conserved miRNAs might have the least contributory role as universal biomarkers but may play roles in more species-specific aspects of disease pathogenesis and outcomes. Apart from the sequence conservation of miRNAs, the presence of the same miRNA signatures in both humans and animals upon contracting the same infectious disease supports the concept of common biomarkers. Taken together, these observations indicate that cross-species comparisons of human and animal miRNA expression profiles as well as their conservation could provide unique opportunities to exploit miRNAs as universal biomarkers and also underline both commonalities and differences in pathology of the same disease in different species.

**Table 1:** **Sequence conservation of selected mature miRNAs in humans and chicken^a^**.

miRNA	Identity (%)	Human and chicken miRNA sequences		Accession number
mir-29a	95.2	Human	UAGCACCAU 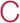 UGAAAUCGGUU 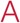	MIMAT0000086
		Chicken	UAGCACCAU 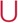 UGAAAUCGGUU 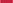	MIMAT0001096
mir-18a-5p	100.0	Human	UAAGGUGCAUCUAGUGCAGAUA 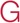	MIMAT0000072
		Chicken	UAAGGUGCAUCUAGUGCAGAUA 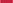	MIMAT0001113
mir-32-5p	100.0	Human	UAUUGCACAUUACUAAGUUGC 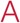	MIMAT0000090
		Chicken	UAUUGCACAUUACUAAGUUGC 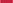	MIMAT0001125
mir-223-3p	100.0	Human	UGUCAGUUUGUCAAAUACCCC 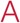	MIMAT0000280
		Chicken	UGUCAGUUUGUCAAAUACCCC 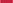	MIMAT0001140
mir-34a-5p	100.0	Human	UGGCAGUGUCUUAGCUGGUUGU 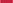	MIMAT0000255
		Chicken	UGGCAGUGUCUUAGCUGGUUGU 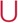	MIMAT0001173
mir-142-3p	100.0	Human	UGUAGUGUUUCCUACUUUAUGG 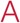	MIMAT0000434
		Chicken	UGUAGUGUUUCCUACUUUAUGG 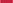	MIMAT0001194
miR-155-5p	100.0	Human	UUAAUGCUAAUCGUGAUAGGGG 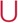	MIMAT0000646
		Chicken	UUAAUGCUAAUCGUGAUAGGGG 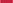	MIMAT0001106

*^a^miRNAs are listed in ascending numerical order. Non-conserved nucleotides are printed in red. Accession numbers of miRNAs sequence are according to MiRBase 21*.

#### Limitations in Using miRNAs as Biomarkers to Combat Viral Diseases

While using miRNAs as novel biomarkers in the veterinary field represents a promising concept, it comes with unique challenges. One challenge is the presence of miRNA isomers (isomiRs), i.e., forms of miRNA that differ slightly from the annotated mature sequence. They are likely created by the enzymatic addition of adenine, cytidine, or uridine and/or imprecise cleavage by the enzymes Dicer or Drosha (21). Observational studies have shown that isomiRs can be regulated upon infection and hence are biologically and functionally meaningful (22). There is some functional overlap between miRNAs and their isomers (23). However, most miRNA annotation tools ignore these isomers by considering them as either noise or sequencing artifacts. The presence of isomiRs might affect miRNA stability and repression capability (24) and therefore reduce their value as biomarkers. When looking for miRNAs as circulating biomarkers, it is important to consider the low miRNA yield (1–10 ng/μl) in body fluids, such as plasma, serum, and urine (25). While some studies suggested that plasma contains a higher miRNA concentration than serum (26), a growing body of evidence has indicated that using serum as a biological sample for miRNA biomarker studies might be biased (27). This is because the stress that blood cells are exposed to during coagulation results in the release of nucleic acids, including miRNAs into the serum, which may change the true repertoire of circulating serum miRNAs giving rise to biased values. With this in mind, the lack of correlation in detection of some miRNAs in plasma and serum is not unexpected. Prior centrifugation of the blood and hemolysis might affect the amount and stability of the target miRNA and require some modifications in the isolation protocols (26). Moreover, difficulties in miRNA extraction can compromise yield and quality (28). Considering that miRNAs are differentially expressed among different animal breeds (29, 30), it is plausible that miRNA levels may differ among different animal breeds if they contract the same disease. This is also apparent among humans where the expression of some miRNAs was found to be related to ethnicity. In this regard, receiver operating characteristic (ROC) curve analysis indicated that let-7c predicted the onset of breast cancer with an area under the curve (AUC) of 0.99 in African Americans while having an AUC of only 0.78 in Caucasians. On the other hand, the best predictor in Caucasians was miR-589 with an AUC of 0.85 (31). This holds true for other biomarkers as well. Two reports documented a significant breed effect on the level of plasma NT-proBNP, a diagnostic marker in dogs with degenerative mitral valve disease (DMVD) (32, 33).

### RNA Interference as a Promising Tool for Therapeutic Intervention

RNA interference is a form of post-transcriptional gene silencing that can function in a broad range of eukaryotic species. Fighting animal viruses with RNAi can be mediated by using siRNA or miRNAs, although the origin of both molecules is different. While miRNAs are endogenously produced throughout two processing steps in nucleus and cytoplasm, siRNA can be exogenously introduced directly into the cytoplasm as a double strand (34). Once in the cytoplasm, both miRNA and siRNA pass through the same processing steps where they are digested by the Dicer enzyme to form a duplex. Only one strand of this duplex is translocated into the RNA-induced silencing complex (RISC) to mediate its function (35). While siRNA forms a perfect complementarity with its target mRNA, causing its cleavage, miRNAs tend to bind to their mRNA targets less perfectly leading to repression in translation. Historically, laboratory-based experiments of using RNAi to block the replication of animal viruses started early on, namely in 2003 against IAV (36). Harnessing miRNAs for therapeutic use will rely on using gain and loss of function and is linked to the expression level of the miRNA (35). miRNAs that are beneficial for the virus and are up-regulated upon infection might be blocked using classic or modified anti-miRNAs (37). In this regard, antagomiRs (cholesterol conjugated anti-miRNAs) have been used *in vitro* and *in vivo* (38). Chemically modified nucleotides, such as locked nucleic acid (LNA), and other modifications have made it conceivable to design more stable and specific oligonucleotides. In an *in vivo* system, reports stated that the effect of using LNA proved to be long-lasting and safe, as neither toxicity associated with LNA nor histopathological changes were detected (39). Although there are attempts to downregulate the Dicer or Drosha enzymes as indirect ways to block miRNAs, this mechanism should be strictly controlled since blocking these enzymes will affect the entire miRNA population (40). In cases where miRNAs tend to inhibit virus replication, a therapeutic approach could be to over-express these miRNAs or to restore their levels. In this context, synthetic miRNA mimics resembling mature miRNAs that could be recognized by RISC would be a suitable tool (40). The *in vivo* delivery of miRNA modalities to specific cells has remained a substantial barrier. Using viruses or virus-like vectors might be innovative approaches since viruses have evolved over many generations to infect certain cells and to deliver foreign RNA, including miRNA, in a tissue- and cell-specific manner (41). Viral vectors can express pri-miRNA or pre-miRNA-like structures or even mature miRNA. Here, RNA viruses of both nuclear and cytoplasmic origin have been utilized (42). miRNAs may have advantages over siRNAs as therapeutic candidates. In spite of having off-target effects, miRNAs bind to their targets with partial complementarity (43) and, thus, likely tackle the high rate of mutation seen in many viruses better than siRNAs. Also, siRNAs can trigger interferon production as part of a cellular stress response pathway that can cause translation arrest, growth inhibition, and cytotoxicity (44). In contrast to the shRNA approach, the use of miRNAs enables the expression of multiple miRNAs from a single transcript as compared to only one in regular shRNA vectors. Indeed, transfection of cells with two different shRNAs may lead to competition of the two for transport and incorporation into the RISC, resulting in a reduction in shRNA processing and activity (45). Despite reports on efficient silencing of genes using RNAi, differences in the efficacy of a given vector between experiments have been reported. This might be due to inefficient cellular uptake of the RNAi and may also depend on the cell type. What follows is an overview and update of the *in vitro* and *in vivo* experiments aiming at evaluating the potential use of small RNAs, including miRNAs, as a treatment option against viral diseases that affect animals of agricultural and/or economic importance.

#### Influenza A Virus

Infection with IAV is a worldwide problem that affects both human and animal health (46, 47). The presence of multiple viral genotypes and the possibilities of antigenic shift and drift continue to raise concerns about the pandemic potential (48, 49). Current influenza vaccines and therapies have proved to be inefficient to combat the continuously evolved IAV strains due to the occurrence of antigenic variation within influenza virus genomes due to point mutations (drift) or re-assortment (shift) (50, 51). The emergence of resistant virus strains added another limitation to anti-IAV therapies (52). RNAi formulated in an appropriate agent would offer the potential for a new therapy by targeting viral transcripts. Furthermore, inserting a let-7b response element within the H1N1 genome created an attenuated strain that conferred protection in mice against challenge with a lethal strain, suggesting that the attenuated strain might serve as a live-attenuated vaccine (53). Around 13,500 possible siRNA target sites are present in the IAV genome. Recent reports described the usefulness of methods and procedures to select highly effective influenza-specific siRNAs in cell culture, mice, and ferrets (54). Using *in silico* approaches, Raza and colleagues identified five conserved amino acid sequences, three in the hemagglutinin (HA) gene (RGLFGAIAGFIE, YNAELLV, and AIAGFIE) and two in the neuraminidase (N) (RTQSEC and EECSYP) gene, which might provide potential RNAi-based therapeutic targets in various IAV strains (55). RNAi has been shown to be effective in suppressing IAV replication both *in vitro* and *in vivo*. For instance, transfecting MDCK cells with siRNA specific for nucleoprotein (NP, nucleotide positions 1496–1514) or polymerase acidic (PA, nucleotide positions 2087–2106) mRNA sequences inhibited IAV replication (36). Moreover, a mixture of siRNAs specific for highly conserved regions of NP and PA can protect mice from lethal challenge with IAV of the H5 and H7 subtypes [e.g., Ref. (56)]. siRNA against the matrix 2 (M2) gene exhibited similar or slightly higher reduction in virus replication in MDCK cells and in human HEK293 cells (57). Likewise, IAV titers in MDCK cells and in embryonated eggs were reduced more than 50- and 100-fold, respectively, when shRNA targeting the polymerase basic 1 (PB1) gene was transfected *in vitro* and *in vivo* using a liposome-encapsulated pSIREN/PB1 vector. In mice, the survival rate ranged between 50 and 100% (58). In another experiment, siRNA targeting a region of the M1 gene between nucleotides 331 and 351 was found to be the most effective in inhibiting M1 protein translation in cell lines. Inhibiting the viral M1 protein using this siRNA caused an 80% reduction in viral titers in supernatants of siRNA-transduced MDCK cells at 6, 8, and 10 hpi. Furthermore, virus budding ability was reduced by 40%, suggesting the ability of siRNA targeting the M1 protein to suppress IAV replication (59). Another report demonstrated the efficacy of anti-NP and anti-PA shRNAs in reducing IAV titers in MDCK cells and in avian CH-SAH cells. Significant decreases of up to 80% in the levels of IAV NP mRNA and up to 370-fold in viral titer were observed in the CH-SAH cells. The approach also worked well in MDCK cells, as demonstrated by significant decreases up to 90% in the level of viral mRNA, and up to 106-fold in IAV infective titer. Furthermore, the authors identified a novel, highly efficient, and conserved RNAi target site in the viral NP gene, which can be used in antiviral cocktails of shRNAs to prevent IAV escape from RNAi silencing (60). Zhou and colleagues investigated the silencing effect of M2 and NP-specific siRNAs on IAV (H5N1, H1N1, and H9N2) replication in cell lines and mice (61). In the cell lines, a 0.51–1.63 TCID_50_ reduction in virus titers was observed, and delivery of pS-M48 and pS-NP1383 significantly reduced lung virus titers in the infected mice (16- to 50-fold reduction in titer) and partially protected them from lethal IAV challenge. As an alternative approach, targeting host cell genes that are crucial for IAV replication can be conducted to control the virus. Expression of α2,3-linked (avian-type) and α2,6-linked (human-type) sialic acid (SA) receptors on host tissues is considered one of the host range and tissue tropism determinants of influenza viruses. An siRNA duplex was used to inhibit IAV binding and internalization *via* silencing *ST6GAL1* gene that encodes the β-galactoside α-2,6-sialyltransferase I (ST6Gal I), a protein important in SA receptor formation (62). In addition, targeting cellular proteases has been discussed as a method to suppress IAV replication. Rogers and colleagues studied pulmonary miRNA expression in mice infected with the IAV H5N1 strain and verified that furin, a member of the convertase family that mediates cleavage of hemagglutinin, is a target gene for miRNAs upon H5N1 infection (63). This highlights the importance of using miRNAs as potential therapeutic agents against IAV.

#### Venezuelan Equine Encephalitis Virus

*Venezuelan equine encephalitis virus* (VEEV) belongs to the genus alphavirus in the family Togaviridae. This virus is still endemic in many parts of the world and is considered an emerging disease threat in other parts as well as a potential biological weapon (64). So far, there are no US Food and Drug Administration (FDA) approved drugs or vaccines against VEEV. Thus, developing artificial miRNAs that can be used to control VEEV infection is a step in the right direction. Indeed, VEEV has been targeted efficiently by siRNA (65). Most recently, it was shown that targeting the viral non-structural protein-4 (nsp-4) region with miRNAs in BHK-21 cells efficiently inhibited viral replication, with artificial miR-3 having the greatest effect (66). This study indicated that these artificial miRNAs merit further testing in animal models for antiviral therapies against VEEV infection.

#### Foot-and-Mouth Disease Virus

Foot-and-mouth disease (FMD) is a highly infectious viral disease that usually affects cloven-hoofed animals. The direct impact of an FMD outbreak includes great losses to agricultural production and disruption of local economies, while the indirect effects lie in the disease control measures at both local and global levels and the high cost of disease control and prevention programs. FMDV has an RNA genome and many serotypes, and targeting conserved viral genes, such as 3D, VP4, and 2B, is a major aim in order to control FMD (67). The use of peptide-conjugated morpholino oligomers (PPMOs) and miRNAs with sequences complementary to various segments of the FMDV genome effectively blocked viral replication in cell culture models (68). Likewise, DNA vector-based RNAi technology can specifically suppress the expression of the VP1, 3D, VP4, and 2B genes and thus inhibit viral replication *in vivo* and *in vitro* (67, 69). Using adenovirus-based vectors to express siRNA molecules in cell lines and mice, Kim et al. suggested to apply RNAi treatments before and after infection with FMDV (70). Treatment after FMDV infection inhibited viral replication effectively, but a combination of treatment before and after infection gave the best results in pig kidney cells, IBRS-2 cells, and in suckling mice, as evidenced by lower viral titers in cell lines and higher survival rates of the treated mice. These experiments did reveal that the RNAi method took considerable time to induce a silencing effect, which ranged from 24 to 48 h (71, 72). This is considered a limitation when attempting to control certain rapidly spreading contagious diseases, including FMD, as viral spread will be faster than the inhibitory action of the RNAi. Finally, the use of artificial miRNAs (amiRs) resulted in specific silencing of reporter genes fused to FMDV target sequences (73).

#### Classical Swine Fever Virus

Classical swine fever virus (CSFV) can cause a hemorrhagic disease in pigs characterized by disseminated intravascular coagulation, thrombocytopenia, and immunosuppression (74, 75). CSFV has been recognized for nearly 200 years and now appears to have been eradicated in Europe and North America due to vaccinations and other control measures. The first study of using siRNA in blocking CSFV replication was conducted in 2008 (76). Three siRNA molecules targeting different regions of the CSFV Npro and NS5B genes were prepared and transfected into PK-15 cells. They caused a 4- to 12-fold reduction in viral genome copy number. In another study, synthetic siRNA transfected into swine kidney cells (SK-6) could target nucleotides 1130–1148 in the nucleocapsid protein (C) of the CSFV with subsequent reduction in viral titer compared to either mock-treated or non-treated cells (77). This emphasizes the potential of siRNA to inhibit CSFV replication. Clearly, *in vivo* experiments need to be conducted to confirm this effect.

#### Rabies Virus

Rabies is a zoonotic disease caused by rabies virus (RV), a member of the Rhabidoviridae family. The disease typically infects canines (78) and is usually transmitted by animal bites, causing a lethal encephalitis. The annual number of deaths due to rabies has been estimated to be approximately 59,000 (79). The control of RV in wild carnivores has moved from culling operations to parenteral and oral vaccination of susceptible species (80), but inhibiting viral replication with siRNA or miRNAs may be another promising approach. Cell lines have been used to assess the usefulness of siRNA in inhibiting RV replication either by using a pool of siRNAs (81) or by single and multiple artificial miRNA targeting RV nucleocapsid (N) (45). In these *in vitro* assays, there was a comparable virus reduction at 72 h post-infection, especially when a single miRNA completely matched the target. Similar results were reported by others [e.g., Ref. (82)]. In cultured cells and murine model, RV glycoproteins were proved to be essential for trans-synaptic viral spread between neurons (83). This observation encouraged other researchers to target the genes encoding such glycoproteins. Sonwane et al. studied the ability of adenovirus-based siRNAs, delivered to BHK-21 cells, to inhibit RV replication and subsequently tested this approach in mice (84). In this study, siRNA inhibited viral replication in cell lines and mice. In BHK-21 cells, siRNA targeting the RV polymerase gene (L gene) was found to be more effective than siRNA targeting the RV NP (N gene) in inhibiting and reducing RV replication. Specifically, a 48.2% reduction of RV foci was seen in cells, in which the L gene was targeted versus a 41.8% reduction when the N gene was targeted. A significant, even greater, difference was observed at the mRNA level (17.8- versus 5.7-fold reduction). In mice, inoculation of both siRNA vectors resulted in a 50% protection against a subsequent lethal RV injection. siRNAs simultaneously targeting the glycoprotein G and N genes led to an 87% reduction in viral release, demonstrating that siRNAs directed against different targets may act synergistically and increase efficacy of siRNA-based interventions against RV (85). Taken together, the above results do suggest that use of siRNAs constitutes a promising approach to interventions against RV.

#### Viral Diseases of Fish

Viral infection in fish aquaculture can be devastating and costly (86). Early reports of RNAi-based treatments described use of this technology in fish and shellfish in 2008 (87). In fish betanodavirus, there are two amino acid residues in the B2 protein (R53 and R60), which bind viral RNA to circumvent the RNAi pathway, underscoring the importance of the antiviral role of the host RNAi machinery (88). Dang et al. showed an inhibitory effect of siRNA on seabream iridovirus, a marine fish virus (89). In this study, siRNA introduced into cells infected with red seabream iridovirus specifically and effectively bound to mRNA encoding the virus major capsid protein, leading to a reduction in the production of virus particles in the supernatant of virus-infected cells, as compared to the cells receiving the control treatment. These results provide encouraging evidence that siRNA technology might be used to control fish viral diseases. More recently, a shRNA construct was found to inhibit the proliferation of viral hemorrhagic septicemia virus by targeting its G gene in a sequence-specific manner (90). Infection with herpesvirus 3 causes severe financial losses in the common carp and koi culture industries worldwide (91). Although most investigations have employed *in vitro* approaches, RNAi might be a promising tool to combat herpesvirus 3 in carp. For instance, a pool of siRNAs specific for DNA enzyme synthesis and capsid proteins of cyprinid herpesvirus 3 virus can be a potential inhibitor of virus replication in carp fibroblasts (92). Along the same line, Gotesman et al. demonstrated that siRNAs can inhibit the thymidine kinase and DNA polymerase genes of cyprinid herpesvirus 3, causing decreased release of viral particles from transfected common carp brain cells (93). Viral infection in shrimp constitutes a great problem, and excellent reviews have discussed the use of RNAi in controlling various viral infections in shrimp [e.g., Ref. (94–96)].

### Potential Use of RNAi to Create Genetically Engineered Virus-Resistant Animals

Genetic selection has been successful in mediating remarkable progress in livestock improvement. Genetic engineering of livestock is commonly used to produce pharmaceuticals or to enhance production characteristics of animals but has also proven to be important in producing animals with infectious disease resistance. For example, cows have been genetically engineered to be resistant against *Staphylococcus aureus*-induced mastitis (97), and laboratory investigations have been conducted with regard to creating α-herpesvirus-resistant livestock (98). Furthermore, there are efforts to create livestock resistant against gastroenteritis coronavirus infection, but published studies are limited to work with mice (99). Against IAV infection, two potent lentivirus-based shRNAs targeting the NP and PA genes of IAV were used to generate IAV-resistant mice (100). However, a successful challenge experiment has not been reported in this system. Subsequent studies based on inhibiting genes of other pathogens have been conducted (61). With improved RNAi techniques, it is conceivable that genetically engineered disease-resistant animals, based on siRNA or shRNA technology, may someday become reality in veterinary infectious disease medicine. Even prion diseases have been the target of transgenic-animal technology featuring shRNAs. Golding and colleagues attempted the use of siRNA technology to generate prion-resistant goat and cattle (101). First, they designed a lentivirus-based shRNA tagged with green fluorescent protein (GFP), which was directed against caprine prion protein precursor (PrP^c^) mRNA and then transfected this vector into an adult goat fibroblast cell line. These cells were then used for somatic nuclear transfer to produce transgenic goat embryos for subsequent *in vitro* differentiation in various stages of pre-implantation development. They confirmed the silencing capacity of shRNA in brain tissue of the growing fetus compared to an age-matched normal fetus. The authors observed an approximate 90% reduction in the expression of PrP^c^. However, clinical efficacy in reducing the risk of a neurodegenerative disease was not determined, and data regarding efficacy were not presented. This suggests that this technique had surpassed a major technical hurdle. Furthermore, two studies described the efficacy of RNAi to silence FMDV in transgenic bovine fetal epithelium cells (BFEC), although rigorous negative controls were lacking, making it difficult to ascribe any effects to the transgenic manipulations. The first of these was conducted by Wang et al., who describe the construction of three recombinant lentiviral vectors containing shRNA against VP2 (RNAi-VP2), VP3 (RNAi-VP3), or VP4 (RNAi-VP4) of FMDV and subsequent testing of their silencing power in both 293 and BHK-21 cells (102). The lenti-RNAi-VP4 vector was transfected into bovine fetal fibroblast cells. The stably transfected cells were transferred into enucleated oocytes, and the reconstructed embryos were then transferred to recipient cows. shRNA expressed in transgenic fetuses significantly degraded viral RNA after inoculation with FMDV at a titer of 100 TCID_50_ and inhibited viral replication. Thus, primary transgenic bovine fetus tongue epithelium cells became much more resistant to FMDV challenge. In the second report, a shRNA-expressing lentiviral vector targeting VP1 of FMDV resulted in strong suppression of VP1 protein expression in 293T cells and also significantly inhibited viral replication in BHK-21 cells (103). The construct was then transfected into bovine fetal fibroblast cells. Cloning these somatic cells resulted in 3-month-old transgenic fetuses. FMDV RNA synthesis and viral replication were significantly reduced in primary tongue epithelial cells from the transgenic fetuses, suggesting that RNAi technology can be potentially used to generate transgenic cattle resistant against FMDV. Taken together, the studies summarized above support the idea that transgenic cloning may prove to be a useful tool to deliver antiviral and anti-prion RNAi to the germ line of animals of veterinary importance, but substantial additional work remains to be done before this technology may demonstrate efficacy in veterinary practice.

## Remaining Challenges

Despite the excitement about utilizing non-coding RNAs to combat animal viral diseases, considerable challenges still need to be overcome before they can be used clinically. Animal breeders tend to rear their flocks in large groups under intensive or semi-intensive husbandry or on large farms. It would be wasteful in terms of money, time, and labor to deliver these expensive molecules on an individual basis. In this case, most veterinarians prefer to use antiviral therapies in a common source bio-vehicle, for instance, food, water, or air, to ensure quick accessibility. We think that using individual miRNA-based therapies will be more practical in special cases, such as the following: race horses, the very expensive parent flocks of chickens and turkeys that are intended for production of specific pathogen-free (SPF) eggs, purebred domestic animals kept as stock for distributing semen for artificial insemination, and cross breeding and improving certain animal traits for meat, milk, or fat production. Controlling contagious viral diseases, for instance, FMDV and IAV, necessitates a rapid intervention strategy to prevent virus spread from one farm to another and from animals to human. In this regard, RNAi that produces the inhibitory effect within 1 or 2 days in cell lines is considered to be insufficient, and a more rapidly operating approach is needed. Another technical challenge is that the excessive levels of the introduced miRNAs can saturate the internal host processing machine for other host small RNAs giving rise to toxicity, pathology, and mortality to the animal under therapy (104). Therefore, the dose of the introduced RNAi-based therapy should be well controlled. The delivery of the RNAi molecule is a key roadblock in this whole process. This is because RNAi molecules are negatively charged and do not penetrate the cell membrane effectively, a step that is necessary for subsequent silencing of mRNAs in the cytoplasm (105). Additionally, they may be quickly excreted, of low stability, non-tissue specific, and may have an inefficient intracellular release (106). Although the delivery of the silencing molecule may be mediated *via* vectors, suboptimal vector selection might reduce the silencing effect. Many delivery systems, such as nanoparticles, cationic lipids, calcium phosphate, antibodies, cholesterol, and viral vectors, have been tested (107). From another perspective, the use of a single RNAi silencing molecule with a low percent match with the target mRNA would lead to a poor target reduction. Possible solutions include either applying only one siRNA which is 100% identical to the sequences of interest or applying more than one siRNA sequence targeting different conserved regions of the target gene. In the case of IAV, spontaneous mutations were estimated to occur at a rate of approximately 1.5 × 10^−5^ per nucleotide per infection cycle (50), suggesting that target sequence mismatches will arise inevitably. Another challenge is to develop a universal RNAi molecule against the same sequence in multiple influenza strains. Some viruses may evolve mechanisms to circumvent the targeting RNAi molecule, either by expressing virus-encoded suppressors or by mutation (108). In order to avoid this, scientists have tried to design RNAi molecules that simultaneously target several sequences within a viral gene (109). In practice, in the fish aquaculture system, RNAi-based therapy have demonstrated some limitations. As a rearing system in some fish farms, the rearing cages are kept floating in the sea or river water, the so-called open sea or river cage aquaculture. Under such system, introducing RNAi molecules into fish feed will allow settlement of the uneaten food, containing the therapy, to the bottom of the water body. This would be ineffective and would also make the feed available to non-target organisms (110). Thus, an alternative improved approach would be to use RNAi in land-based ponds or tanks, owing to their direct accessibility to fish and the easy disposal of waste materials. The commercial field application of injectable therapy is neither practical nor realistic, especially with shrimp, which are reared in an intensive system. Despite its relatively high expense, soaking the shrimp in a solution containing the RNAi silencing molecule is a more practical way to ensure that an effective suppression of the gene is achieved (111). Unfortunately, there are no shrimp cell lines available for the research community, delaying a better understanding of the RNAi application in shrimp farms. Effective design of the RNAi molecule is also of special concern. Although various computational tools have been developed to systematically evaluate the targets for miRNAs and or siRNA (112–114), non-specific off-target effects need to be anticipated. The many parameters that influence specificity of miRNAs/siRNAs include the selected target region, size, the starting nucleotide, GC content, the thermodynamic properties of the introduced molecule, and the presence of internal repeats. Apart from an effective design, the use of accurate positive and negative controls is necessary to ensure the validity of RNAi data (115).

## Future Directions

From the evidence gathered thus far, we have every reason to be optimistic about the future use of sncRNAs in the diagnosis, monitoring, and treatment of animal viral diseases. Zoonotic viruses continue to pose a public health threat to humans. There are miRNAs that are associated with zoonotic viral diseases that were found to be conserved among the human and animal reservoirs and exhibit similar tissue tropism. It is import to investigate both the contribution of these miRNAs to the zoonotic nature of diseases and their potential roles as biomarkers or therapeutic tools for humans and animals. This is even more important for viral diseases affecting poultry populations that are reared under both intensive and semi-intensive systems, where the pathogens can be transmitted in a short time to populate the environment and infect susceptible hosts. Regarding the use of RNAi in combating viruses, the search for a target sequence conserved across strains is of highest priority in studies targeting animal viruses, in particular, those featuring rapid genomic changes, such as IAV and other RNA viruses. However, using a pool of various siRNAs or a cocktail of siRNAs specific for virus and host genes might reduce escape of mutant viruses. In addition, it would be valuable to develop more rapidly acting RNAi technology to inhibit spread of highly contagious infections, such as FMD. Prospectively, incorporating the RNAi molecule into animal feed or the water supply might be a practical choice for the treatment of animals reared in large numbers, such as fish or poultry. Using this strategy, successful experiments have been recorded in shrimp infected with white spot syndrome virus (WSSV) (116). In spite of the extensive efforts toward formulating a suitable vehicle, one that delivers the smallest RNAi quantity in a non-toxic way remains to be discovered. In this respect, the use of a natural exosome or a natural or synthetic high-density lipoprotein (HDLP) is a novel and promising approach. These are just a few areas of research that are likely to engage veterinary scientists and virologists for years ahead. These and other improvements should further facilitate the use of miRNA and siRNA to prevent and control animal viruses at veterinary clinical sites and in the field.

## Conclusion

**Figure 2:**
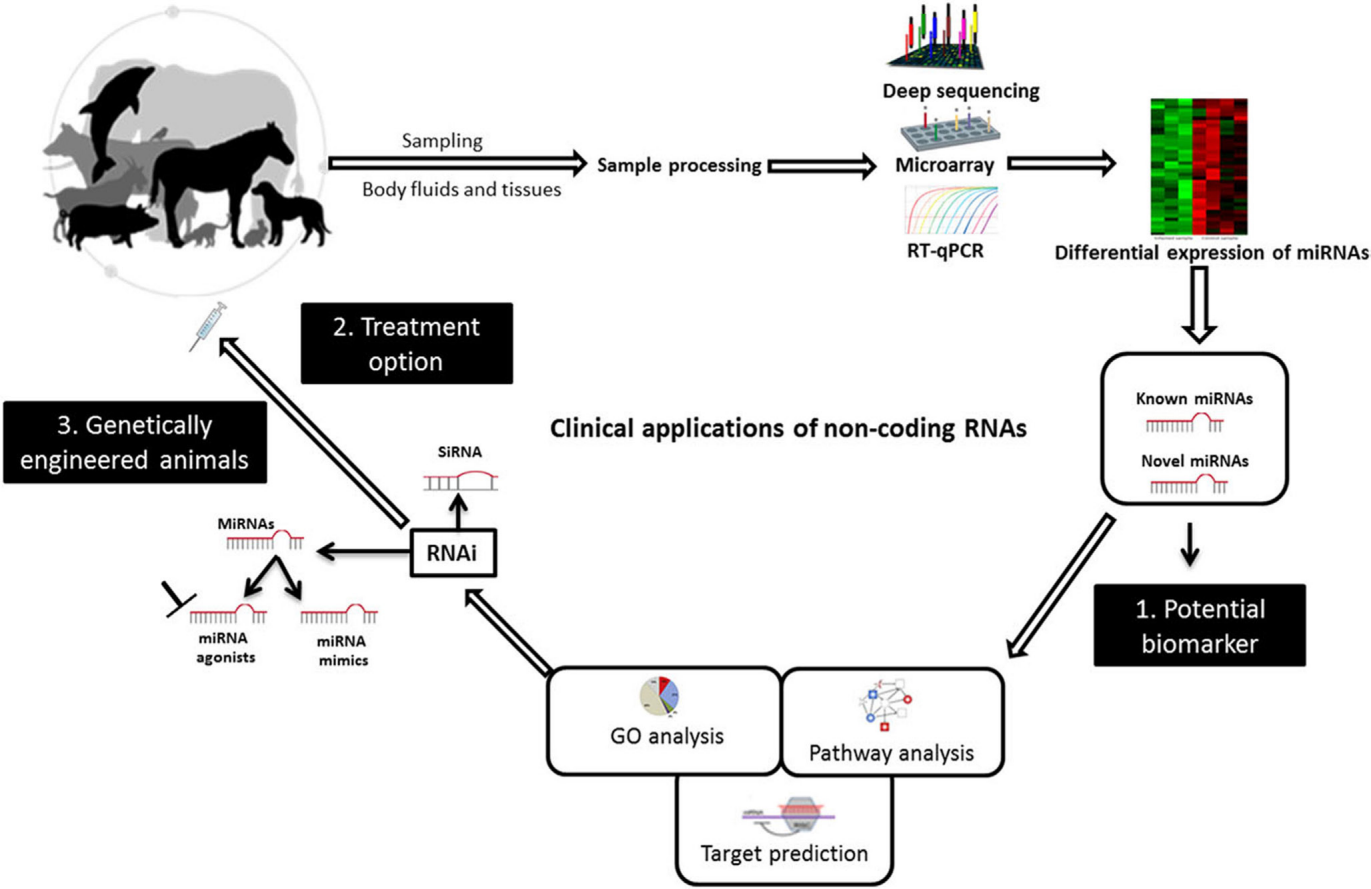
**Diagrammatic illustration of potential uses of non-coding RNAs to combat animal viruses**. miRNAs can be used as biomarkers for pinpointing animal viral diseases. They can act as potential therapies and to create genetically virus-resistant animal breeds. Abbreviations: RT-qPCR, reverse transcriptase quantitative real-time PCR; RNAi, RNA interference; siRNA, small interfering RNA; GO, gene ontology. Adapted from UGA Veterinary Diagnostic Laboratories, Werner et al. (117), and Livingston et al. (118).

Small non-coding RNAs have been known as crucial regulators of gene expression, and they have great potential for applications in the diagnosis, prevention, and treatment of viral infectious diseases of veterinary importance. Research efforts are continuing to translate RNAi technology with its two arms, miRNAs and siRNA, to clinical applications in veterinary medicine (Figure 2). In this respect, the deregulation of miRNAs upon infection, their stability, and tissue specificity have made their study as biomarkers a fruitful area of research. siRNA molecules together with miRNA mimics or agonists can be delivered to the infected animal as a treatment option. Although there are currently no genetically engineered virus-resistant animals, the likelihood of exploiting RNAi technology, including miRNAs, is growing and is expected to help attain this aim. Bringing these molecules to the market will remain to be challenging and many barriers still need to be overcome. In fact, *in vitro* models would enable more detailed studies on the clinical relevance of these molecules. However, experimental animal models and infections of natural hosts in laboratory investigations will afford more realistic insights into the best ways to utilize sncRNAs to improve animal health. Importantly, developing animal-specific databases that contain experimentally validated small RNA molecules and related functional analysis will facilitate using these data for future research. The continual emergence of zoonotic viruses warrants effective collaborations between physicians and veterinarians in this issue. The available evidence suggests that the clinical use of sncRNAs in combating animal viruses may be possible in the not too distant future.

## Author Contributions

MS did the literature search, wrote the initial draft of the manuscript, and prepared the figures and tables. FP oversaw the project, edited the manuscript including the final version, and takes responsibility for the integrity of the data.

## Conflict of Interest Statement

The authors declare that the research was conducted in the absence of any commercial or financial relationships that could be construed as a potential conflict of interest.

## Abbreviations

APPs, acute phase proteins; AUC, area under the curve; BFEC, bovine fetal epithelium cells; CSFV, classical swine fever virus; DMVD, degenerative mitral valve disease; FDA, Food and Drug Administration; FFPE, formalin-fixed, paraffin-embedded tissue; FMD, foot-and-mouth disease; FMDV, foot-and-mouth disease virus; GFP, green fluorescent protein; HA, hemagglutinin; HDLP, high-density lipoprotein; IAV, influenza A virus; LNA, locked nucleic acids; miRNA, microRNAs; PA, polymerase acidic; PB-1, polymerase basic 1; PPMOs, NP, nucleoprotein peptide-conjugated morpholino oligomers; PrP^c^, prion protein of cell; RISC, RNA-induced silencing complex; RNAi, RNA interference; ROC, receiver operating characteristic; RV, rabies virus; SA, sialic acid; shRNA, short hairpin RNA; siRNA, small interfering RNA; sncRNAs, small non-coding RNAs; SPF, specific pathogen-free; UTR, untranslated region; VEEV, *Venezuelan equine encephalitis virus*; WSSV, white spot syndrome virus.
